# *Syzygium jambos* and *Solanum guaraniticum* Show Similar Antioxidant Properties but Induce Different Enzymatic Activities in the Brain of Rats

**DOI:** 10.3390/molecules18089179

**Published:** 2013-07-31

**Authors:** Gabriela Bonfanti, Paula Rodrigues Bitencourt, Karine Santos de Bona, Priscila Sabino da Silva, Letícia B. Jantsch, Aline S. Pigatto, Aline Boligon, Margareth L. Athayde, Thissiane L. Gonçalves, Maria Beatriz Moretto

**Affiliations:** 1 Postgraduate Program in Pharmacology, Department of Clinical and Toxicology Analysis, Health Science Center, Federal University of Santa Maria (UFSM), Santa Maria, RS 97105-900, Brazil; E-Mails: gabriela_bonfanti@yahoo.com.br (G.B.); ksbona@yahoo.com.br (K.S.B.); pry_sabino@hotmail.com (P.S.S.); letii_jantsch@hotmail.com (L.B.J.); thissianegoncalves@yahoo.com.br (T.L.G.); 2 Postgraduate Program in Pharmaceutical Sciences, Health Science Center, Federal University of Santa Maria (UFSM), Santa Maria, RS 97105-900, Brazil; E-Mails: bitencourt.paula@yahoo.com.br (P.R.B.); alineboligon@hotmail.com (A.B.); margareth@smail.ufsm.br (M.L.A.); 3 Franciscan University Center (UNIFRA), Santa Maria, RS 97010-032, Brazil; E-Mail: alinepi@unifra.br

**Keywords:** *Solanum guaraniticum*, *Syzygium jambos*, δ-aminolevulinate dehydratase, lipid peroxidation, induced oxidative stress

## Abstract

*Syzygium jamb*os and *Solanum guaraniticum* are both employed in Brazil as medicinal plants, even though their potential toxicity is not well established and they are frequently misused. The aim of this study was investigate the effect of the aqueous leaf extracts of both plants on δ-aminolevulinate dehydratase (δ-ALA-D) and acetylcholinesterase (AChE) activities and the antioxidant action against oxidative damage induced by sodium nitroprusside in rats, using *in vitro* assays. In addition, the presence of gallic, caffeic and chlorogenic acids, as well as rutin, quercetin and kaempferol as bioactive compounds in the extracts was identified by HPLC and their levels quantified. The antioxidant activities of both extracts were assessed by their capabilities to scavenge nitric oxide and to inhibit lipid peroxidation. Only *Syzygium jambos* presented thiol-peroxidase-like activity. Although neither extract affected the AChE activity, the aqueous extract of *Solanum guaraniticum* inhibited brain δ-ALA-D activity, suggesting a possible impairment effect on the central nervous system. Our results showed that both extracts exhibited efficient free radical scavenger activity and are an interesting source of bioactive compounds, justifying their use in folk medicine, although *Solanum guaraniticum* extract could have neurotoxicity properties and we therefore suggest that its use should be restricted to ensure the health of the population.

## 1. Introduction

Free radicals are accepted as important mediators of tissue injury in several human diseases, such as arthritis inflammation, atherosclerosis, diabetes, cirrhosis and cancer [1]. The efficiency of the antioxidant defense system is altered under pathological conditions and thus, the ineffective scavenging process and/or overproduction of free radicals may play a crucial role in causing tissue damage [2]. Therefore attention has been focused on the search for natural antioxidants with the potential to scavenge free radicals and enhance the antioxidative defense system [3,4].

Medicinal plants have been traditionally used in the treatment of several human diseases and their pharmacological and therapeutic properties have been attributed to the different chemical constituents isolated from their crude extracts [5]. As an example, the leaves of *Syzygium jambos* (L.) Alston (Myrtaceae) have used as a diuretic, in the treatment of rheumatism, as a febrifuge and present antiviral, anti-inflammatory and digestive properties [6,7,8]. In Brazil this plant is known by the common name “jambolão” and its leaf infusions are also used traditionally in the treatment of diabetes, even thugh some studies have shown its ineffectiveness as a antihiperglycemic agent [9]. Likewise, *Solanum guaraniticum* A. St.-Hil (Solanaceae) is known by the folk name “falsa-jurubeba” in southern Brazil. Its traditional uses include the treatment of anemia, fevers and liver and gastric dysfunctions such as hepatitis and ulcers [10,11,12]. However, although previous studies have demonstrated the hepatoprotective and antioxidant activities of aqueous extract of *Solanum guaraniticum*, they also showed that it promoted an increase in the level of serum hepatic enzymes in mice and it is related to cattle intoxications [13,14,15]. Thus, although medicinal plants may have biological activities that are beneficial to humans, the potential toxicity of these bioactive substances has often not been well established [16]. In particular, data on the toxic effects of Brazilian *Syzygium jambos* and *Solanum guaraniticum* are scarce and they are often misused in folk medicine.

The enzyme δ-aminolevulinate dehydratase (δ-ALA-D, EC 4.2.1.24) is a sulfhydryl-containing enzyme of heme pathway [17]. Due to its active sulfhydryl groups and the role of heme proteins in many cellular metabolic processes [18], the enzyme presents high sensitivity to pro-oxidant situations and impairment of metabolic processes and has been used to evaluate toxic effects [19,20]. Another important enzyme present in all animals, acetylcholinesterase (AChE, EC 3.1.1.7) is important for function of the cholinergic system, by hydrolysis of the neurotransmitter acetylcholine [21]. The crucial role of cholinesterases in neural transmission makes them a primary target of a large number of cholinesterase-inhibiting drugs and toxins [22], and making them valuable diagnostic tools for verifying exposure to chemical agents [23].

The purpose of this study was to evaluate the effects of aqueous extracts of *Solanum guaraniticum* and *Syzygium jambos* on δ-ALA-D and AChE activities. The effects of both extracts on the lipid peroxidation, thiol status and catalase activity on induced oxidative stress in tissue of rats were assessed using *in vitro* assays.

## 2. Results and Discussion

### 2.1. Pytochemical Screening of Aqueous Extract of *Syzygium jambos* and *Solanum guaraniticum*

**Figure 1:**
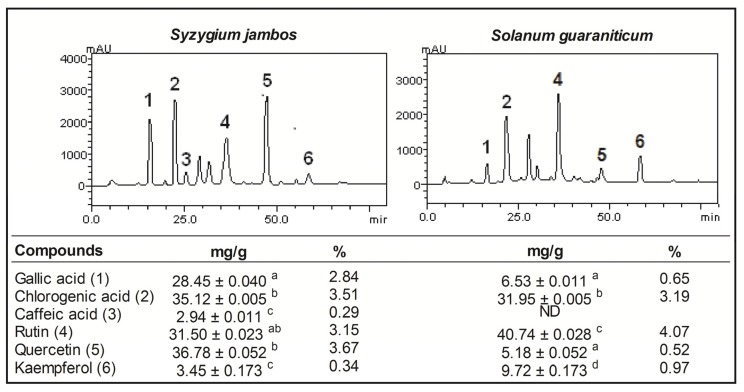
HPLC/DAD profile of extracts tested (detection UV at 271 nm) and quantification of phenolic compounds found [Gallic acid (peak 1), chlorogenic acid (peak 2), caffeic acid (peak 3), rutin (peak 4), quercetin (peak 5) and kaempferol (peak 6). Quantification results are expressed as mean ± SEM (*n* = 3) assessed by one way ANOVA followed by Duncan Multiple Comparison *post hoc* test. Means marked with different letters are significantly different (*p* < 0.05). ND = not detected.

In order to ascertain the phytochemicals responsible for the *in vitro* biological activities of *Syzygium jambos* and *Solanum guaraniticum*, the aqueous leaf extracts of these plants were screened for various compounds. HPLC fingerprinting revealed the presence of gallic and chlorogenic acids, as well as rutin, quercetin and kaempferol in both extracts and the percentage of each compound is shown in Figure 1. Furthermore, considering the chromatographic conditions used, caffeic acid was only detected in the *Syzygium jambos* extract. The characterization of these aqueous leaf extracts is particularly important since there is scarce data available about this local species and any new studies represent a significant contribution to the knowledge of these plants [7,24].

The contents of total phenol, flavonoids, and vitamin C are also shown in Table 1. As can be seen, *Syzygium jambos* aqueous leaf extract has a higher content of total phenolic compounds (*p* = 0.0002) whereas *Solanum guaraniticum* extract has higher total flavonoids content levels (*p* < 0.0001). This result agrees with the proportion of phenolic acids found in the HPLC analysis of the extracts (Figure 1). Along this line, *Solanum guaraniticum* extract has higher levels of vitamin C (*p* = 0.0010) than *Syzygium jambos* extract. As an electron donor, vitamin C is a potent water-soluble antioxidant in humans and an essential dietary nutrient required as a co-factor for several enzymes [25,26]. However, the human body lacks the ability to synthesize this compound, therefore the vegetal species with high content of vitamin C are valuable sources of this nutrient. Furthermore, vitamin C has been reported to contribute to the antioxidant activities of plant food and is present substantially in the extracts. Ascorbic acid is a good reducing agent and exhibits its antioxidant activities by electron donation [27].

Polyphenols are considered to be strong antioxidants due to the redox properties of their hydroxyl groups [28]. Phenolic compounds are capable of removing free radicals, chelating metal catalysts, activating antioxidant enzymes, reducing a-tocopherol radicals, and inhibiting oxidases [29].

**Table 1:** Total phenols, total flavonoids and vitamin C contents in aqueous extracts of *Syzygium jambos* and *Solanum guaratiticum.*

Extract content	*Syzygium jambos*		*Solanum guaraniticum*	
Total phenolic (mg GAE/g)	108.2 ± 3.34	58.76 ± 1.72 **		
Total flavonoid (mg QE/g)	85.55 ± 2.54	237.90 ± 7.12 ***		
Vitamin C (mg VIT C/g)	21.07 ± 0.64	58.01 ± 4.21 **		

Data are reported as mean ± SEM (*n* = 3). GAE: gallic acid equivalents; QE: quercitin equivalents; VIT C: vitamin C. Statistically significant differences as determined by Student’s *t*-test. (**) and (***) denotes *p* < 0.001 and *p* < 0.0001.

### 2.2. Thiol Peroxidase-Like Activity of Both Extracts Evaluated

**Figure 2:**
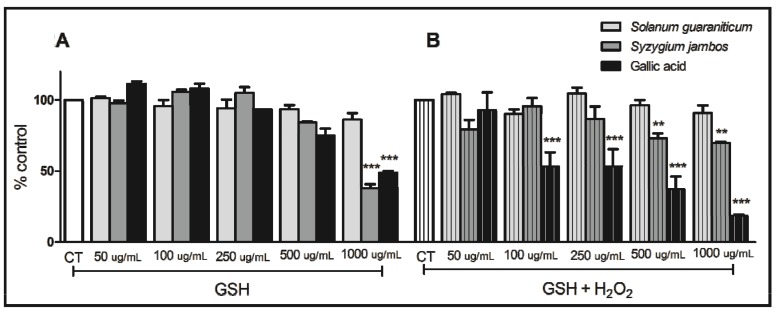
Thiol peroxidase-like activity of aqueous extracts tested in the absence of H_2_O_2_ (**A**), and in the presence of H_2_O_2_ (**B**). Data are reported as mean ± SEM (*n* = 3) and assessed by one way ANOVA followed by Duncan Multiple Comparison *post hoc* test. (**) and (***) denotes *p* < 0.001 and *p* < 0.0001, compared to respective controls (CT).

In Figure 2B, we can observe a decrease in SH groups when *Syzygium jambos*, at 500 µg/mL and 1,000 µg/mL, was incubated with hydrogen peroxide (H_2_O_2_). Considering this effect, *Syzygium jambos* seems to catalyze the reduction of H_2_O_2_ with the consumption of glutathione (GSH), thus mimicking the properties of glutathione peroxidase (GPx). GPx is an antioxidant selenoenzyme that protects various organisms from oxidative damage by catalyzing the reduction of H_2_O_2_ and other organic peroxides with the help of GSH as the reducing agent [30]. Taking into account that mimetic activities of this enzyme have been investigating in synthetic compounds to explore their antioxidant capacity [20], this results give us an insight into the possible mechanisms by which *Syzygium jambos* extract exerts its effects, which are under investigation in our laboratory. 

However, we also noticed that *Syzygium jambos* extract at 1,000 µg/mL was able to promote the consumption of GSH even in the absence of H_2_O_2_ (Figure 2A), which might have occurred due to a possible reaction between thiols and extract components or GSH oxidation. In fact, plant extracts can present a pro-oxidant or antioxidant behavior depending on the dose [31]. However, in the presence of H_2_O_2_, due to a likely competition of this reactive species and GSH as well as due to the extract components, the polyphenol extracts may be used to detoxify H_2_O_2_. Moreover, *Solanum guaraniticum* extract did not cause any changes on SH groups level, neither in the presence nor in the absence of H_2_O_2_ suggesting that this extracts, at the concentrations tested, did not present thiol-peroxidase like activity such as antioxidant capacity.

### 2.3. Nitric Oxide-Scavenging Activity Assay of Extracts

**Figure 3:**
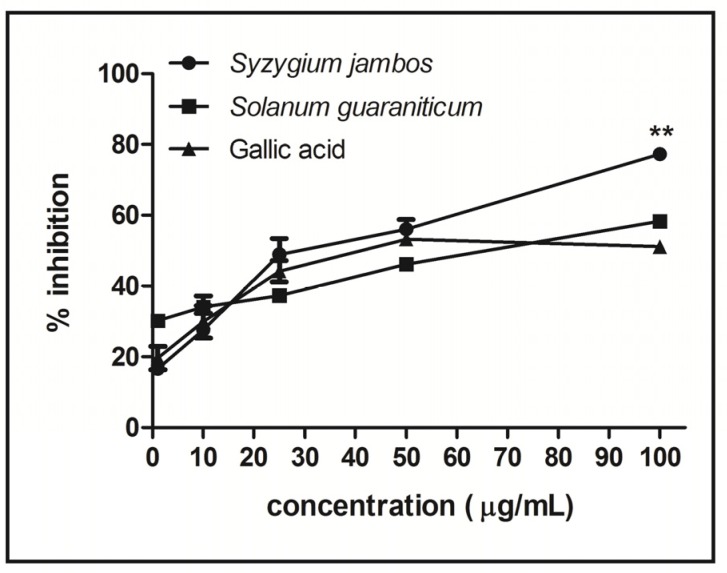
NO scavenging activity of aqueous extracts tested. Data are reported as mean ± SEM (*n* = 3) and assessed by one way ANOVA followed by Duncan Multiple Comparison *post hoc* test. ** denotes *p* < 0.001 compared to *Solanum guaraniticum* and gallic acid (100 µg/mL).

Figure 3 shows that both aqueous extracts studied can scavenge nitric oxide (NO) radical, but *Syzygium jambos* had a significantly higher scavenging ability than *Solanum guaraniticum*. At the higher concentration tested (100 µg/mL), the scavenging capacity of *Syzygium jambos* extract was around 77%, while *Solanum guaraniticum* demonstrated a scavenging power around 58% (*p* < 0.001).

### 2.4. Effect of Extracts on δ-ALA-D and AChE Activity in Rat Tissues

In this study, *Solanum guaraniticum* extract inhibited brain δ-ALA-D activity at 1,000 µg/mL (*p* < 0.05). Taking into account that: (i) some studies have related *Solanum guaraniticum*, under its synonym *S. fastigiatum*, to bovine intoxications affecting the central nervous system of the animals and causing neuronal degeneration and sporadic seizures [13,14,32]; and (ii) δ-ALA-D activity can be inhibited during seizures [33]; this finding may be considered an indication of the toxic properties of this plant. Furthermore, the inhibition of the δ-ALA-D causes an accumulation of its substrate 5-aminolevulinic acid (ALA), which has already demonstrated neurotoxicity by inducing seizures and death after intracerebroventricular administration in rodents [34]. ALA may also rapidly oxidize to generate reactive oxygen species [35], which could intensify the toxicological process. Free radicals are one of the main causes of cellular dysfunction in the brain [36], and seizures, oxidative stress and δ-ALA-D activity have already been suggested as linked events [37]. On the other hand, δ-ALA-D activity of liver and kidney tissues was not affected by *Solanum guaraniticum* extract. *Syzygium jambos* was not able to inhibit δ-ALA-D activity of any tissue tested (Table 2). Furthermore, none of the concentrations of any extracts tested was able to alter enzymatic activity of AChE (Table 2).

**Table 2:** Effect of extracts on tissue δ-ALA-D and AChE activity in rat homogenates.

Treatment	δ-ALA-D			AChE
	Liver	Brain	Kidney	Brain
Control (PBS)	4.33 ± 0.48	1.23 ± 0.18	1.49 ± 0.43	2.78 ± 0.14
Lead acetate 10 µM	2.92 ± 0.23	0.95 ± 0.02	0.99 ± 0.43	-
Paraoxon 1 µM	-	-	-	1.26 ± 0.07 ***
*Syzygium jambos* 100 µg/ mL	4.85 ± 0.71	1.10 ± 0.06	1.89 ± 0.49	2.65 ± 0.14
*Syzygium jambos* 250 µg/mL	4.09 ± 0.46	1.38 ± 0.28	1.97 ± 0.53	2.75 ± 0.14
*Solanum guaraniticum* 500 µg/mL	4.24 ± 0.52	0.91 ± 0.11	1.66 ± 0.32	2.73 ± 0.14
*Solanum guaraniticum* 1000 µg/mL	4.31 ± 0.72	0.66 ± 0.14 *	1.35 ± 0.18	2.79 ± 0.14

Data are reported as mean ± SEM (*n* = 6) and assessed by one way ANOVA followed by Duncan Multiple Comparison *post hoc* test. δ-aminolevulinate dehydratase activity (δ-ALA-D) results are presented as nmol porphobilinogen (PBG)/mg protein/h. Acetylcholinesterase actitivty (AChE) results are presented as µmol AcSCh/h/mg of protein. (*) and (***) denotes *p* < 0.05 and *p* < 0.0001 as compared to the respective control samples.

### 2.5. Effects of *Syzygium jambos* and *Solanum guaraniticum* on Lipid Peroxidation, NPSH Content and Catalase Activity of Sodium Nitroprusside (SNP)-Induced Tissues

The ability of both studied aqueous extracts to inhibit SNP-induced lipid peroxidation was measured by the production of thiobarbituric acid reactive substances (TBARS) and the results are presented in Table 3. The data revealed that the incubation of the tested homogenates in the presence of SNP caused a significant (*p* < 0.05) increase in TBARS content (484.75% to liver, 911.01% to brain and 295.06% to kidney) when compared with the basal value (100%). However, the presence of aqueous extract of *Syzygium jambos* or *Solanum guaraniticum* at all concentrations tested inhibited TBARS production, with a more pronounced effect in the brain tissue. 

**Table 3:** Effect of aqueous extracts of *Syzygium jambos* and *Solanum guaraniticum* on lipid peroxidation level before and after sodium nitroprusside (SNP) incubation of rat homogenates.

Treatments	Liver	Inhibition (%)	Brain	Inhibition (%)	Kidney	Inhibition (%)
Control	2.23 ± 0.54	-------	3.36 ± 0.93	-------	1.62 ± 0.57	-------
Gallic acid 25 µg/mL	1.26 ± 0.42	43.49	1.57 ± 0.51	53.27	1.29 ± 0.13	20.37
*Syzygium jambos* 100 µg/mL	1.98 ± 0.52	11.21	1.53 ± 0.18 *	54.46	0.93 ± 0.25	42.59
*Syzygium jambos* 250 µg/mL	2.01 ± 0.51	9.86	1.19 ± 0.26 **	64.58	0.73 ± 0.18	54.93
*Solanum guaraniticum* 500 µg/mL	2.36 ± 0.53	-------	1.49 ± 0.26 *	55.65	0.81 ± 0.15	50.00
*Solanum guaraniticum* 1000 µg/mL	2.41 ± 0.40	-------	1.89 ± 0.35 *	43.75	0.84 ± 0.14	48.14
Induced (SNP)	10.81 ± 1.45 ***	-------	30.61 ± 3.41 ***	-------	4.78 ± 1.47 *	-------
Gallic acid 25 µg/mL	1.08 ± 0.09 ^###^	90.00	27.88 ± 1.15	8.91	1.30 ± 0.22 ^###^	74.26
*Syzygium jambos* 100 µg/mL + SNP	2.39 ± 0.51 ^###^	77.89	2.97 ± 0.61 ^###^	90.29	1.09 ± 0.30 ^##^	77.19
*Syzygium jambos* 250 µg/mL + SNP	2.33 ± 0.47 ^###^	78.44	1.95 ± 0.78 ^###^	93.62	0.86 ± 0.10 ^##^	82.00
*Solanum guaraniticum* 500 µg/mL + SNP	2.58 ± 0.65 ^###^	76.13	1.86 ± 0.80 ^###^	93.92	1.00 ± 0.20 ^##^	79.07
*Solanum guaraniticum* 1000 µg/mL + SNP	2.75 ± 0.59 ^##^	74.56	2.77 ± 0.74 ^###^	90.95	1.18 ± 0.17 ^##^	75.31

Data are reported as mean ± SEM (*n* = 6) and assessed by one way ANOVA followed by Duncan Multiple Comparison *post hoc* test. Results are presented as nmol MDA (malondialdehyde)/mg protein. (*), (**) and (***) denotes *p* < 0.05, *p* < 0.001 and *p* < 0.0001, respectively, as compared to the respective control samples. (#), (##) and (###) denotes *p* < 0.05, *p* < 0.001 and *p* < 0.0001, respectively, as compared to the respective induced samples.

These findings are in accordance with the NO scavenging effect of *Syzygium jambos* and *Solanum guaraniticum* verified in Figure 3. SNP cause cytotoxicity through the release of cyanide and/or nitric oxide (NO) [38]. Therefore, a plausible mechanism by which these extracts are conferring protective action against SNP-induced lipid peroxidation could be the ability of the extract phytochemicals to scavenge NO radicals produced by SNP. The phenolic compounds in the extracts can also quench free radicals which may have been resulted from lipid peroxidation chain reaction [39]. In fact, phenolics present in plant sources have received considerable attention over the past decade because of their potential to prevent lipid peroxidation and diseases associated with it [40]. In line with this, we can associate the antioxidant activity of extracts studied here with their folk medicinal use. *Syzygium jambos* leaves are often used to manage diabetes mellitus, a disease for which the involvement of lipid peroxidation is well known [41]. *Solanum guaraniticum* is also used mainly to treat liver diseases and the lipid peroxidation process can contribute to the initiation and progress of liver damage [42].

The non-protein thiol (NPSH) status of the tested homogenates was also verified. Table 4 shows that the extracts did not affect thiol status of tissues alone and that the presence of SNP decreased thiol levels (56.63% to liver and 65.13% to brain tissue) when compared with the basal value, except for kidney tissue. However, both *Syzygium jambos* and *Solanum guaraniticum* extracts, at all concentrations tested, were capable of maintaining thiol level at the brain homogenate when they were present in the SNP incubation.

**Table 4:** Effect of aqueous extracts of *Syzygium jambos* and *Solanum guaraniticum* on non-protein thiol (NPSH) content and catalase activity in the liver, brain and kidney of rats.

Treatments	Thiol content			CAT		
	Liver	Brain	Kidney	Liver	Brain	Kidney
Control	11.16 ± 2.16	7.57 ± 1.01	5.66 ± 1.19	38.14 ± 6.49	6.36 ± 1,49	17.71 ± 2.22
Gallic acid 25 µg/mL	11.44 ± 2.6	9.85 ± 0.29	7.31 ± 0.78	40.28 ± 9.87	6.34 ± 0.34	26.53 ± 1.53 *
*Syzygium jambos* 100 µg/mL	7.41 ± 0.84	9.12 ± 0.50	6.21 ± 1.81	37.65 ± 7.81	4.16 ± 1.25	15.41 ± 2.75
*Syzygium jambos* 250 µg/mL	7.32 ± 1.89	10.66 ± 0.73	9.73 ± 1.36	28.72 ± 6.54	2.80 ± 1.10	13.84 ± 2.33
*Solanum guaraniticum* 500 µg/mL	6.42 ± 1.82	9.20 ± 1.00	10.52 ± 1.76 *	41.98 ± 6.18	3.43 ± 1.07	20.80 ± 2.03
*Solanum guaraniticum* 1000 µg/mL	10.94 ± 2.35	10.91 ± 1.69	8.84 ± 0.20	49.10 ± 6.45	3.37 ± 1.17	18.82 ± 1.65
Induced (SNP)	4.84 ± 1.69 *	2.64 ± 0.57 **	5.42 ± 1.70	46.78 ± 9.98	4.04 ± 1.17	16.74 ± 1.01
Gallic acid 25 µg/mL	5.51 ± 0.57	3,01 ± 0.20	5.43 ± 0.54	53.47 ± 10.44	2.95 ± 0.56	26.52 ± 3.08 ^#^
*Syzygium jambos* 100 µg/mL + SNP	5.80 ± 1.46	7.52 ± 0.79 ^##^	6.13 ± 1.00	48.04 ± 7.70	4.13 ± 1.42	17.92 ± 1.52
*Syzygium jambos* 250 µg/mL + SNP	6.66 ± 0.90	9.40 ± 0.65 ^###^	10.56 ± 1.96 ^#^	40.04 ± 9.82	2.37 ± 1.37	19.47 ± 3.23
*Solanum guaraniticum* 500 µg/mL + SNP	8.45 ± 1.87	7.30 ± 0.52 ^##^	6.95 ± 0.62	47.69 ± 13.39	4.17 ± 1.01	19.18 ± 1.17
*Solanum guaraniticum* 1000 µg/mL + SNP	7.96 ± 1.67	9.03 ± 0.86 ^###^	9.50 ± 1.51	41.60 ± 11.14	3.52 ± 1.06	19.18 ± 1.69
Data are reported as mean ± SEM (*n* = 6) and assessed by one way ANOVA followed by Duncan Multiple Comparison *post hoc* test. Results are presented as mmol NPSH (non-protein thiol)/mg protein and units of catalase (CAT)/mg protein. (*) and (**) denotes *p* < 0.05 and *p* < 0.001, respectively, as compared to the respective control samples. (#), (##) and (###) denotes *p* < 0.05, *p* < 0.001 and *p* < 0.0001, respectively, as compared to the respective induced samples.						

The brain and nervous system are particularly vulnerable to oxidative stress due to their limited antioxidant capacity [43]. In this context, the thiol redox state is an essential parameter associated with major biologic processes such as oxidative stress and intracellular redox homeostasis [44]. In fact, the results observed in Table 4 demonstrated that brain NP-SH level was more sensitive to SNP and more responsive to both extracts tested than the liver and kidney. This effect could be linked to the phytochemical compounds identified on the extracts since several studies of polyphenol rich foods or individual flavonoids have demonstrated their neuroprotective properties against oxidative and inflammatory stressors [45].

In relation to enzymatic antioxidants, the SNP treatment was not able to alter significantly the CAT activity, even the extracts alone, in any of the tissue tested. Similar results were observed in previous studies [20,46]. Catalase is a heme protein that catalyzes the reduction of H_2_O_2_ and protects tissue from highly reactive hydroxyl radicals [47]. Thus, the effect observed here may have occurred because this enzyme is not directly effective towards the reactive species formed on the *in vitro* system.

Previous studies have already demonstrated the radical scavenging properties and antioxidant effect of *Solanum guaraniticum* on ferrous-induced lipid peroxidation [24]. In relation to *Syzygium jambos*, radical scavenging properties and *in vivo* antioxidant power have already been demonstrated [48] although this is the first study involving the aqueous extract of this plant, which is its most popular form of medicinal use [9]. In this sense, a deep evaluation of the antioxidant activity by different methods is substantial for a better understanding of biological potential activities of plant extracts, since the distinct antioxidant properties could indicate that they were acting via distinct mechanisms. 

## 3. Experimental

### 3.1. Chemicals and Apparatus

5'-Aminolevulinic acid (δ-ALA), thiobarbituric acid (TBA), 5,5'-dithiobis(2-nitrobenzoic acid) (DTNB), gallic acid, ascorbic acid and quercitin were purchased from Sigma (St. Louis, MO, USA). Sodium nitroprusside (SNP) was obtained from Merck (Darmstadt, Germany). All other chemicals were of analytical grade and obtained from standard commercial suppliers. High performance liquid chromatography (HPLC-DAD) was performed with a Shimadzu HPLC system (Shimadzu, Kyoto, Japan), comprising a Prominence Auto Sampler (SIL-20A), equipped with Shimadzu LC-20AT reciprocating pumps connected to a DGU 20A5 degasser, CBM 20A integrator and SPD-M20A UV-VIS diode array detector (DAD) and Software LC solution 1.22 SP1. Absorbance measurements were recorded on a Hitachi U-18,000 UV-Visible Reading Spectrophotometer (Hitachi High-Technologies Corporation, Tokyo, Japan) using disposable cuvettes for the visible range, and quartz cuvettes for measurements in the ultraviolet (UV) range.

### 3.2. Plant Material and Preparation of Extracts

Leaves of *Solanum guaraniticum* and *Syzygium jambos* were collected in the cities of Boca do Monte and Tupanciretã, respectively. A voucher specimen was identified and deposited at the herbarium of Federal University of Santa Maria. The leaves were dried in a greenhouse, smashed in a knife mill and submitted to extraction with ethanol 80% in a Soxhlet apparatus until exhaustion. After extraction, the solvent was evaporated on a rotavapor, supplying the crude extract. 

### 3.3. Phytochemical Analysis

#### 3.3.1. High-Performance Liquid Chromatography (HPLC) Characterization

HPLC characterization under gradient conditions using C_18_ column (4.6 mm × 250 mm) packed with 5 μm diameter particles; the mobile phases were water containing 2% acetic acid (A) and methanol (B), and the composition gradient was: 5% of B until 2 min and then changed to obtain 25%, 40%, 50%, 60%, 70% and 100% B at 10, 20, 30, 40, 50 and 80 min, respectively. The flow rate was 0.7 mL/min, injection volume 40 μL and the detection wavelengths were 271 nm for gallic acid, 325 nm for caffeic and chlorogenic acids, and 365 nm for quercetin, rutin and kaempferol. The chromatography peaks were confirmed by comparing their retention times with those of reference standards and by DAD spectra (200 to 500 nm). 

#### 3.3.2. Total Polyphenols Content

Total phenolic content of extracts (1 mg/mL) was determined with Folin-Ciocalteu’s reagent in alkaline medium and expressed as milligram of gallic acid equivalents per gram of extract powder (mg GAE/g) [49].

#### 3.3.3. Total Flavonoid Content

The total flavonoid content, as milligram of quercitin equivalents per gram of extract powder (mg QE/g), was measured on extracts (1 mg/mL) based on aluminum chloride colorimetric method reported by Zhishen and Mengcheng [50].

#### 3.3.4. Determination of Vitamin C Content

The vitamin C content was determined in extracts (1 mg/mL) using the method of Benderitter *et al*. [51], based on its reaction with 4-dinitrophenylhydrazine (DNPH), calculated using a vitamin C standard curve and expressed as milligram of vitamin C per gram of extract powder (mg VIT C/g).

### 3.4. Nitric Oxide-Scavenging Assay of Extracts

The scavenging effect of extracts on nitric oxide (NO) was measured according to the method of Sreejayan and Rao [52]. Gallic acid was used as positive control. For the assay, sodium nitroprusside (10mM), was mixed with different gallic acid or extracts concentrations, incubated 150 min and then mixed with 0.5 mL of Griess reagent and measure at 546 nm. In the control, sample extract was substituted by PBS. The capability of scavenging NO was calculated using the following equation:

Scavenging effect (%) = [1 − (Asample/Acontrol)] × 100
(1)

### 3.5. Thiol Peroxidase-Like Activity of Extracts

The catalytic effect of *Solanum guaraniticum* and *Syzygium jambos* on the reduction of hydrogen peroxide (H_2_O_2_) by reduced glutathione (GSH) was assessed using the rate of GSH oxidation. Different concentrations of extracts were incubated in the medium containing GSH (1mM) with and without H_2_O_2_ (0.3 mM). At 120 min, aliquots of the reaction mixture (200 µL) were checked for the amount SH groups according to Ellman [53]. Gallic acid was used as positive control. The values are expressed in percentage of control [20].

### 3.6. Animals

Male adult albino Wistar rats (200–250 g) from our own breeding colony, that are maintained at 22 ± 2 °C, on a 12 h light/dark cycle, with water and food were provided *ad libitum*. The animals were used according to the guidelines of the Committee on Care and Use of Experimental Animal Resources, Federal University of Santa Maria, Brazil (Process number 23081.009003/2012-01). All efforts were made to minimize the number of animals used and their suffering. The rats were euthanized and the brain, liver and kidney tissues were rapidly dissected, weighted and placed on ice. The tissues were immediately homogenate in 10 mM Tris-HCl, pH 7.4 (1/10 *w*/*v*). The homogenates were centrifuged at 4,000× *g* at 4 °C for 10 min to yield a low-speed supernatant (S1) that was used for δ-ALA-D and AChE activity besides TBARS, thiol content and catalase activity assays. Moreover, protein content of S1 was measured by Peterson [54].

3.6.1. δ-ALA-D Activity

δ-ALA-D activity was assayed by the method of Sassa [55] with some modifications. An aliquot of S1 (200 μL) was incubated at 37 °C in the presence or absence of aqueous extracts at different concentrations (100 and 250 µg/mL of *Syzygium jambos* and 500 and 1,000 µg/mL of *Solanum guaraniticum*), based on previous studies in our laboratory. Enzymatic reaction was initiated by adding the substrate δ-aminolevulinic acid and the incubation was carried out at 37 °C for 1 h to liver and kidney and for 3 h to brain homogenate. The porphobilinogen which is formed during the incubation period, was mixed with modified Ehrlich’s reagent, and the color developed was measured spectrophotometrically (555 nm) against a blank. A set of tubes was assessed with lead acetate (AcPb, 10 µM) as positive control of inhibition enzymatic activity [56] Results were expressed as nmol porphobilinogen (PBG)/mg protein/h. 

#### 3.6.2. Acetylcholinesterase Activity Assay for Brain

The AChE enzymatic assay was determined by a modification of the spectrophotometric method of Ellman *et al.* [57] as previously described [58]. An aliquot of brain homogenate (50 μL) was pre-incubated for 1 h at 37 °C in the presence or absence of aqueous extracts at different concentrations (100 and 250 µg/mL of *Syzygium jambos* and 500 and 1,000 µg/mL of *Solanum guaraniticum*). The reaction was initiated by adding 0.8 mM acetylthiocholine iodice (AcSCh) to the reaction mixture (2 mL final volume) contained 100 mM TFK; pH 7.5 and 1 mM 5,5'-dithio-bis-nitrobenzoic acid (DTNB). The method is based on the formation of the yellow anion measured by absorbance at 412 nm during 2-min incubation at 25 °C. Paraoxon (1 µM, an organophosphate inhibitor of AChE), was used as positive control [59]. The enzyme activity was expressed in µmol AcSCh/h/mg of protein. 

#### 3.6.3. Sodium Nitroprusside (SNP) induced Oxidative Stress

This assay was carried out to determine if the extracts protect against oxidative stress induced by SNP in rat brain, liver and kidney homogenates *in vitro*. SNP was used as classical inductor of oxidative stress. The supernatant of each tissue was incubated with or without freshly prepared SNP (50 µM) and different concentrations of the plants extract (100 and 250 µg/mL of *Syzygium jambos* and 500 and 1,000 µg/mL of *Solanum guaraniticum*) at 37 °C for 1 h. After the incubation time, lipid peroxidation was measure by TBARS levels according to the method of Niehaus and Samuelsson [60], nonprotein thiols (NPSH) content was determined according to Ellman [53] and catalase activity as described by Aebi [61]. Gallic acid 25 µg/mL was used as positive control in all assays [5].

### 3.7. Statistical Analysis

The analyses were performed using STATISTICA for Windows, version 6.0 (StatSoft. Inc., Tulsa, OK, USA). All data were analyzed using Student’s *t*-test or one way ANOVA, followed by Duncan’s multiple range test, when appropriate and presented as mean ± standard error of mean (SEM). A value of *p* < 0.05 was considered statistically significant for all analyses. 

## 4. Conclusions

The study of medicinal plants is important, in particular because the population generally believes that plant extracts are safe due their natural origin, although studies have shown that even plants used popularly with therapeutic purposes can present different degrees of toxicity. The current study has unequivocally demonstrated the antioxidant effect of both aqueous leaf extracts tested, evidencing properties to justify their popular use, meanwhile, *Solanum guaraniticum* extract inhibited the brain δ-ALA-D activity, suggesting a possible impairment on the central nervous system. The results presented in this study indicate that *Solanum guaraniticum* may be neurotoxic and caution must be exxercised in its administration. Therefore, more studies are needed to clarify its mechanisms of action.
